# Components of stigma and its impact on maternal and child health service and outcomes: perspective of Akha hill tribe women in Thailand

**DOI:** 10.1186/s12913-022-08622-x

**Published:** 2022-10-19

**Authors:** Thanatchaporn Mulikaburt, Tawatchai Apidechkul, Pilasinee Wongnuch, Siwarak Kitchanapaibul, Anusorn Udplong, Peeradone Srichan, Panupong Upala, Chalitar Chomchoei, Fartima Yeemard, Ratipark Tamornpark, Onnalin Singkhorn

**Affiliations:** 1 School of Health Sciences, Mae Fah Luang University, Chiang Rai, Thailand; 2 Center of Excellence for Hill Tribe Health Research, Mae Fah Luang University, Chiang Rai, Thailand; 3 School of Nursing, Mae Fah Luang University, Chiang Rai, Thailand; 4 School of Health Science, Mae Fah Luang University, 333 Moo 1, Ta Sud Subdistrict, Muang District, 57100 Chiang Rai Province, Thailand

**Keywords:** Akha, Hill tribe, Stigma, Experience, Maternal and Child Health Care

## Abstract

**Background:**

Maternal and child health (MCH) is crucial to the well-being of mothers and children. Stigma regarding access to MCH services is a major challenge, especially for hill tribe people in Thailand. The study aimed to understand the components of stigma and its impact on MCH service and outcomes including experiences and expectations to address the stigma in perspective of Akha hill tribe women in Thailand.

**Methods:**

A phenomenological qualitative approach was used to gather information from Akha women who had attended MCH service one year prior and had an experience with stigma. A validated question guide was used in the study. The interview was conducted in private and confidential rooms in the Akha hill tribe villages between June and September 2021. A thematic analysis was used to extract the major and minor themes and develop the findings.

**Results:**

A total of 61 Akha postdelivery participants were recruited to provide information; the average age was 28.9 years, 32.8% had no Thai ID card, and 93.4% were married. Language, traditional clothing, poverty, and name were identified as drivers of stigma, while health care providers’ background, gender differences between clients and health care providers, and knowledge gaps facilitated the stigma. Being a member of a hill tribe acted as the stigma marker. Stigma manifestation was presented in the forms of verbal or physical abuse, refusal to provide treatment, and intentional disclosure of personal information to the public. Accepting the situation with no better option, defending oneself to receive better care and services, and using a private care service were experiences in addressing the stigma. Gender matching, active MCH service, mobile emergency clinics, and appropriate, permanent medical equipment in health care facilities located in their villages were the expectations.

**Conclusion:**

Akha women face a variety of stigmas in access to MCH services, with substantial impacts on health outcomes, especially the rate of services in women and child health. Creating laws to prevent the occurrence of any forms of stigma and implementing gender matching in MCH services should be considered.

**Supplementary Information:**

The online version contains supplementary material available at 10.1186/s12913-022-08622-x.

## Background

Stigma is defined as one of the major barriers to accessing health care services [1–4]. Moreover, its impact is greater among vulnerable populations [5, 6]. Maternal health and child care (MCH) is a significant service to people globally to ensure that both mothers and children have good health, including promoting survival during the pregnancy, childbirth and postnatal periods [7]. In 2017, the World Health Organization (WHO) reported that 295,000 women died during and following pregnancy and childbirth [8]. The WHO also recommends that in all stages of MCH, care should minimize negative experiences to ensure that women and their infants reach their full potential of health and wellbeing, especially among vulnerable populations [7]. The major negative experience among pregnant women is stigma [9]. The stigma that women encounter during their visits to MCH services could minimize the rate of access to MCH [10, 11]. Several impacts have been reported from the stigma in access to MCH services, such as poor child health [9] and human immunodeficiency virus (HIV) infection [12]. Many illnesses related to unable to access MCH are required a large amount of money for the treatment and care, especially in developing countries [13, 14], including Thailand [15]. Furthermore, a number of illnesses related to women’s reproductive health can lead to premature death and reduce quality of life [16–18].

In Thailand, MCH services are provided to all reproductive groups and children, including those who do not hold Thai identification (ID) cards, which are used for access to all public services [19]. Commonly, all health institutes provide an MCH service every Tuesday, including a small, so-called health-promoting hospital located in the hill tribe villages [20]. At the health-promoting hospital, pregnant women are cared for under the guidelines of the Thai Ministry of Public Health, including an assessment of general health and detection of potential risks [21]. However, one study in Chiang Rai, Thailand, reported that only 7.1% received three doses of tetanus toxoid during pregnancy, and less than 50% of pregnant hill tribe women accessed MCH services properly [22].Moreover, 64.3% of Akha pregnant women gave birth at home by untrained midwives, and only 30% of Akha children received vaccines based on Thailand expanded program on immunization (EPI) program [23].

The presence of stigma when attending a clinic exerts the greatest impact on certain populations with specific characteristics [24–26]. The hill tribe people in Thailand have moved down from South China over several centuries [27]. There are six main tribes: Akah, Lahu, Hmong, Yao, Karen, and Lisu [27]. Akha people comprise the largest group, with their own culture, lifestyle, and language [27] which is different from Thai people [28]. Most Akha in Thailand live under the national poverty line [29] and have poor education [24]. Inaddition, Akha people are very limited in their use of the Thai language [30]. While all health caregivers are Thai then it is difficult to Akha people to access the services [31].

The stigma present for minority groups when attending health care institutions is well recognized [32]. In the current study, a health stigma and discrimination framework was used as a guideline for understanding the enacted stigma that exists, which is the form of the stigma perceived by individuals [33] while accessing MCH services among Akha women [5]. According to Stangl et al. [5] concept of stigma, a stigma driver is the factor that drives stigma presentation in a phenomenon, and some factors work to facilitate stigma presentation, while the stigma marker is the original marker for the existing stigma. Understanding the component of stigma present for hill tribe women when access MCH services can be applied for health policy formulation and public health implementation to reduce the stigma. Minimizing the stigma encountered by hill tribe women seeking MCH services could improve their access to all clinical services related to women’s health, including screening for cervical and breast cancer. Reducing the stigma encountered during health care services, especially in an MCH service, will improve both the quality and quantity of the services.

The study aimed to understand the components of stigma and its impact on MCH service and outcomes including experience and expectation to address the stigma in perspective of Akha hill tribe women in Thailand.

## Methods

### Study design and setting

A phenomenological qualitative approach [34] was used to elicit information from participants who were Akha hill tribe women living in seven hill tribe villages located along the Thailand-Myanmar border who experienced stigma while accessing MCH services. Akha women who were pregnant or had delivered their child one year prior to data collection and had accessed an MCH service at least once were invited to participate in the study.

### Research tool and its development

The questions were developed from a review of the literature, information obtained from health care providers who worked in the hill tribe villages, and from some pregnant women who had experienced stigma while attending an MCH service. The validity and reliability of the questions were tested before use in the field. Three external experts who were public health professional, medical anthropologist, and nurse working at MCH services were invited to validate the question information and the research context and content. The objective of the validity test was to confirm that the contents of the questions covered the context required in the study. The questions were piloted among six postdelivery women who lived in two hill tribe villages in Mae Chan District, Chiang Rai Province, Thailand. The main objective of the pilot test was to ensure that both the researchers and participants understood the same meaning and sense of the questions provided. Ultimately, seven questions were finalized for use in the study: (1) Which hospital did you attend for MCH services? (2) Did you experience any discomfort or stigma when attending MCH services? (3) Can you provide information in terms of frequency, who displayed stigmatizing behaviors, and in what form? (4) How did you feel about this experience? (5) How did you respond to these behaviors? (6) What is your expectation about accessing MCH services? (7) Did you experience other barriers to accessing MCH services?

### Sampling and recruitment

Village headmen were informed about the study and asked to select participants five days in advance according to the inclusion criteria. The participants were purposively selected from seven hill tribe villages. Hill tribe women who were postdelivery one year prior who had experienced stigma when attending an MCH clinic and who able to use Thai met the inclusion criteria. Women who met the criteria were informed by the village headman and asked to participate in the study. At the date of the interview, women who met the criteria and intended to provide information to the researcher were screened again to determine whether they had evidence according to the criteria. Only those who had a strong experience with stigma were invited to an interview. All participants were provided with information about the study and signed written consent forms that stated the voluntary nature of participation. Three researchers who were trained in qualitative methods (one female medical anthropologist (Ph.D.), one female health behavioral scientist (Ph.D.), and one male public health expert (Ph.D.)) and working as university faculty were the interviewers. All interviewers were women who were familiar from previous projects with the hill tribe people living in these areas.

### Data collection

Face-to-face interviews were conducted in a private and confidential room at the community hall in each village between June and September 2021. A question guide was used in the interview. Before the interview, the participants were asked for permission to record it and take field notes. The interviews started with the objective of research and general questions about maternal and child health. The specific questions on asking about the experience of stigma while attending MCH services were followed. Each interview lasted for 45 min. All methods were carried out in accordance with the Declaration of Helsinki [35] in the ethical principles for medical research that involve human subjects.

### Data analysis

All records were transcribed and checked before further analysis. The transcript was sent to all participants who were the information owner to check its accuracy before further analysis. The information in the transcripts was coded, and coding trees were developed. The codes were transferred into the NVivo program (NVivo, qualitative data analysis software; QSR International Pty Ltd., version 11, 2015) for theme extraction. A content analysis was used to extract major and minor themes with the inductive method, which usually uses the keywords presented from interviews to construct the themes. The major theme was used to present the form or pattern of the stigma while attending MCH services. The minor theme was focused on the other significant surrounding information, including the experiences in addressing the stigma, and expectations of the participants to further solve the problem. All themes identified were constructed and formed. Significant quotations were presented to support the findings.

### Rigor and trustworthiness

**Fig. 1:**
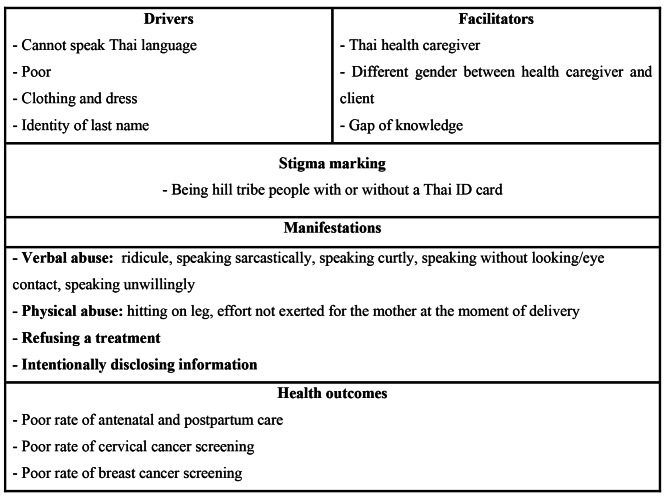
Stigma frame among hill tribe women who access MCH services

Before deciding on final interpretations, the researchers once again sent the information back to the participant who was the information owner to ensure the accuracy of the final findings. Two qualitative research experts in the field were asked to validate the final findings and framework (Fig. 1). The final framework was discussed and validated again with eight local Akha people (five women and three community leaders).

## Results

### General characteristics of the study population

A total of 61 postdelivery women were recruited into the study; the average age was 28.9 years, 32.8% had no Thai ID cards, and 93.4% were married. More than half were Buddhist (55.7%), 67.2% had completed either primary or high school, 59.0% were unemployed, and 44.3% had no regular income. Less than half had been screened for cervical cancer (31.1%) and breast cancer (21.3%) in the previous year (Table 1).

**Table 1:** General characteristics of the participants

	n	%
**Total**	61	100.0
**Age** mean age = 28.9 years, min = 18, max = 41		
**Identification card** (ID card)		
No	20	32.8
Yes	41	67.2
**Marital status**		
Married	57	93.4
Ever married	4	6.6
**Religion**		
Buddhist	34	55.7
Christian	27	44.3
**Education**		
No education	15	24.6
Primary school	16	26.2
High school	25	41.0
Diploma	3	4.9
Bachelor’s degree	2	3.3
**Occupation**		
Unemployed	36	59.0
Daily wage job	13	21.3
Farmer	9	14.8
Merchant	3	4.9
**Income** (bath)		
No income	27	44.3
≤1,000	1	1.6
1,001–5,000	15	24.6
5,001–10,000	12	19.7
≥10,001	6	9.8
**Number of children**		
mean = 2.3, min = 1, max = 9		
**Number of pregnancies**		
mean = 2.4, min = 1, max = 12		
**Had been screened for cervical cancer one year prior**		
No	42	68.9
Yes	19	31.1
**Had been screened for breast cancer one year prior**		
No	48	78.7
Yes	13	21.3

### Components of stigma

The component of stigma consisted of the stigma driver, stigma facilitator, stigma markers, and stigma manifestations. Several factors were clearly identified as drivers, facilitators, markers, and manifestations of stigma for Akha women seeking MCH services. Different levels of the health outcome impact were reported (Fig. 1).

#### A) drivers of stigma for akha women seeking MCH services

Being members of Akha hill tribe with specific culture, as presented through their language, clothing, poverty, and name, was identified as a driver of stigma for Akha women attending MCH services. Most Akha women spoke Thai as a second language, which is completely different from their native language, and only a few Akha women could speak Thai fluently. Most Akha hill tribe women were not supported in school during their youth compared to men, and limited Thai fluency was very common. Therefore, language issues were one of the drivers of the stigma that the Akha women encountered when attending MCH services.

A 30-year-old woman said the following [P#08]:

When I was waiting for delivery, I saw a hill tribe woman next to me being scolded by a nurse after she screamed because she had great pain from the contractions. I truly felt disappointed with the situation. I understood that being a tribe member could cause trouble in communication with Thai people (nurse). I thought, why did a nurse blame her? The nurse should empathize with us.

A 34-year-old woman said the following [P#47]:

If I did not respond to a doctor, it was not because I did not understand what he said, but I could not speak in Thai.

The Akha hill tribe women still favored their traditional cultural clothing, which is different from that of Thai people, and Thai people worked as the main health care providers in the hospital. The Akha hill tribe women’s style of dress differentiated them from other people who attended the clinic and was viewed as non-Thai by others, which was identified as being a driver of the stigmatizing experience.

A 38-year-old woman said the following [P#30]:

I am Akha, and I always wear my traditional costume, including at the time to see the doctor. In addition, I have been looked down on by hospital staff. It might be different from the other (Thai people), but I love to dress in my own traditional style. I feel I am lower than them.

A large proportion of Akha hill tribe people live in poor economic situations, which is a major consequence of being poorly educated and having scant access to skilled jobs. The Akha hill tribe women identified not being able to speak Thai, dressing in their traditional clothing, and living in a poor family as major drivers of stigma when attending MCH services. Poverty was a strong image for health care providers and was identified as one of the greatest drivers of stigma among Akha hill tribe women attending MCH services.

A 28-year-old woman said the following [P#25]:

Due to being the hill tribes, we were blamed by a nurse, and the blaming statements that I always hear are ‘I don’t understand you, what you said. Why do you have more and more children? I told you many times to get sterilization, but you won’t.’ All of these (statements) make me feel truly uncomfortable.

Even though some Akha hill tribe women had been educated in Thai schools, were able to speak Thai fluently, and dressed in a modern style, some of them still used their local tribal name, which easily identified them as hill tribe people. Thus, the name provided to health care providers when attending MCH acted as one of the drivers of the stigma encountered by the Akha hill tribe women.

A 26-year-old woman said the following [P#17]:

At first, the staff did not say anything. She treated me like other people until she saw my surname. She knew automatically that I’m a hill tribe (member). She changed her actions, including speaking with a negative tone. She ordered me to stand away from her and her eyes looked me up and down.

#### B) facilitators of stigma for akha women seeking MCH services

Several factors were identified as facilitators of the stigma encountered by Akha hill tribe women attending MCH services, specifically, the health care providers’ background, gender differences between clients and health care providers, and knowledge gaps.

Most health caregivers were Thai and had different cultures and lifestyles with the Akha hill tribe people. Thus, when the Akha hill tribe women attended MCH services, they were treated based on the health care provider’s background, and some practices hurt or embarrassed the Akha hill tribe women. Moreover, the differences in culture, lifestyle and background between health care providers and clients made it easy to misunderstand communications when receiving services.

A 30-year-old woman said the following [P#31]:

When I went to give birth to my first child, I didn’t know what things I had to prepare for the situation. The hospital staff asked me, ‘You do know you are giving birth, so why do you not prepare all the things you need? Are you going to use hospital supplies?’ The way she spoke to me made me feel guilty and ashamed. I didn’t know how to respond to her at that time. I felt so bad, and it hurts me even now.

A 34-year-old woman said the following [P#47]:

I feel that sometimes, the MCH staff were not happy to serve me. I knew from the words spoken by a nurse. Additionally, a doctor said to me that there were many patients, he didn’t have time to serve only me. I truly understand that the staff have to spend lots of time with us, who don’t know Thai. I wish I could understand and speak Thai fluently.

One additional factor that acted as a facilitator of the stigma encountered by Akha hill tribe women attending MCH services was the gender difference between health care providers and clients. Such differences reduced Akha hill tribe women’s comfort and willingness to attend MCH services.

A 39-year-old woman said the following [P#53]:

When I went to give birth to my second child at a hospital a year ago, a male medical student came to see me while giving birth. It made me feel tense, embarrassed and lacking privacy. In my culture, a man should not get involved in a woman’s delivery, even her husband. It’s not an easy thing to deal with this.

A 31-year-old woman said the following [P#55]:

I had a cervical cancer screening a few months ago. Unexpectedly, the doctor was a male, so I did not want to go to check things anymore. I felt embarrassed. I prefer a female doctor. If I see him again, I won’t know how to behave.

The knowledge gap between clients and health care providers was identified as a significant facilitator of stigma for the Akha hill tribe women attending MCH services. Different knowledge related to care and practice during pregnancy and postdelivery was found to facilitate stigma. In general, the Akha hill tribe women engaged in practices based on their own traditional methods to care for themselves during pregnancy and postdelivery, which might run counter to the knowledge and desirable practices proposed by health care providers. Such discrepancies facilitated the experience of stigmatization. The Akha hill tribe women’s poor knowledge also led to a lack of understanding about how to use their rights to access MCH services under the national universal coverage scheme, which was also a stigma facilitator.

A 32-year-old woman said the following [P#07]:

Anytime I go to see a doctor, I have been treated like I know nothing. Of course, I do not know what he said. For instance, ‘cervical,’ what does it look like? and how’s it important to me? We know nothing, especially medical terms. Additionally, we hill tribe women never attended a school, so then how can we know about that?

#### C) stigma markers for akha women seeking MCH services

Being a member of a hill tribe, with or without holding a Thai ID card, was viewed as a source of stigma in the Akha hill tribe women’s access to MCH services. Being part of a hill tribe was clearly viewed by health care providers as marking these women as “other.” Moreover, being an individual without a Thai ID card was reported as generating stigma.

A 31-year-old woman said the following [P#03]:

I have been viewed as ‘the other’ for all my life. This is because I am a hill tribe (member). Regardless of whether you have a Thai ID card, you are still a hill tribe (member). I have been facing the inequity of health care services since I was born. They speak to me not ‘to inform’ but ‘to order,’ unlike other Thai people.

#### D) manifestations of stigma for akha women seeking MCH services

Several manifestations of stigma were identified by the Akha hill tribe women attending MCH services such as verbal abuse, physical abuse, a refusal to provide treatment, and the intentional disclosure of personal information to the public.

Verbal abuse was very commonly reported among the Akha hill tribe women when attending MCH services. The Akha hill tribe women faced improper, unhealthy, and uncaring speech from health care providers.

A 22-year-old woman said the following [P#05]:

My bad experience happened when I went to a hospital with my one-month-old daughter. I was waiting for (the doctor), and I sat in the wrong chair. Then, a nurse asked me to move to another place. She used an inappropriate voice with me. I was thinking about why she did not speak to me with many polite words. I realized that it was because I am a hill tribe (member).

A 38-year-old woman said the following [P#49]:

Last year, I went to an MCH clinic at a hospital. The staff asked me to explain my problem. I responded to them in my way, but it might not be clear to them. The staff shouted at me with angry responses.

Some Akha hill tribe women experienced physical abuse when attending MCH services, such as being hitting on the leg and nurses not caring appropriately for mothers at the moment of delivery.

A 40-year-old woman said the following [P#16]:

During my first childbirth, a nurse forced me to deliver by telling me to push...push and push. I didn’t understand what push is. Therefore, I did not follow the instructions. A few seconds later, a nurse slapped my legs. I cried and still wonder why they did this to me.

Refusing to give treatment was also reported among the Akha hill tribe women while attending MCH services.

A 26-year-old woman said the following [P#21]:

When I went to work in another province, I chose to go to a health care center near my workplace. When I was there, the staff told me that I did not have the right to receive the services at that center and blamed me for why I was so careless. I felt very sad and asked myself, am I a human? Why cannot the hill tribe people have rights? Why can’t the hill tribe people get health services at other places like the Thai people?

Some Akha women reported that their private information was disclosed, especially to hired staff from the same village as the clients. Once individual information is released to the public, this violation reduces women’s trust in the service and makes them less likely to pursue further care and treatment.

A 25-year-old woman said the following [P#45]:

The staff did not care about me. She did not care about my personal information. I had a cervical cancer screening once, and the staff wanted more women in the village to get this screening, so she took me as an example. She told my personal information to others. I am not OK with this.

#### E) health outcomes

Due to the experience of stigma faced by the Akha hill tribe women when attending MCH services, many poor health outcomes were reported, such as poor rates of antenatal and postpartum care, breast cancer screening (21.3%), and cervical cancer screening (47.5%).

A 41-year-old woman said the following [P#26]:

When I had my third child, I had a very bad experience during delivery at a hospital. With ineffective communication between me and a nurse in the process of antenatal care (ANC), I was ignored in careful care and delayed care. Resulting, I had a large blood bleeding or postpartum hemorrhage.

A 27-year-old woman said the following [P#14]:

I had a serious experience with access and care during my first pregnancy. I was asked to wait for the delivery for a long time, and they (nurses) even carefully cared for me during the waiting. Finally, I found that my baby was in the breech position, and I was transferred to a tertiary hospital for operation. I have a big question to the nurse and doctor that if I was not attending at a hospital close to a tertiary hospital, what would have happened to me?

### Experience in addressing the stigma

After discussing Akha hill tibe women experiences of stigma in many forms, they reported their adaptations in addressing the problem in different ways.

#### Accepting the situation with no better option

The first group of Akha hill tribe women reported that they did not have a better option to access MCH services. Some people reported being provided with poor service but being able to accept the suffering caused by mistreatment. Most impacted Akha hill tribe women accepted their suffering due to the fear of being treated even more poorly the next time.

A 34-year-old woman said the following [P#20]:

“I did not have any response when the staff made me feel uncomfortable or feeling angry by shouting at me or looking down on me. I accepted everything, as I knew I am a hill tribe (woman) who needs help from them. In addition, only they can help me. If I respond to them in bad way, I am afraid that they will not care well for me and my child.”

#### Defending oneself to receive better care and services

Some people preferred to stand up to mistreatment and voice their needs. Their responses to health care providers took different forms, such as reminding the health care providers of their responsibilities, asking the reasons why their case was being ignored, and asking for further information regarding their needs.

A 25-year-old woman said the following [P#06]:

When my first child was born, I remembered that a staff member expressed their negative emotion to me. She tried to talk to me many times, and I felt like I received bad service from this hospital. I asked them to treat me better in their practice. I promise myself that if I have my next child, I won’t come to this hospital.

A 33-year-old woman said the following [P#23]:

A staff member spoke to me impolitely. She told me how to feed my baby. I asked her many times to make sure that I understood the right things. However, at the end, she shouted at me, ‘Don’t you understand?’ I responded immediately, ‘You have your responsibilities to me, or do you want me to complain to your director?’ Then, she changed her emotion and spoke to me nicely.

#### Using a private health care service

Some Akha hill tribe women preferred to obtain services from a private hospital. Generally, at a private hospital, most services are provided based on need. The service mindset of health care providers at private hospitals was perceived as much better than that at public hospitals, which could be due to the impacts of policy and organizational advantages.

A 31-year-old woman said the following [P#40]:

My personal opinion, if I have a lot of money, I prefer to get health care services from a private hospital. The staff will not look down on me.

A 34-year-old woman said the following [P#47]:

Money can help everything. If I had enough money, I would not go to a public hospital but rather to a private hospital without facing stigma.

### Expectations

Regarding their expectations of MCH services, the Akha hill tribe women requested gender-matched providers in MCH services, especially for Papanicolaou test (PAP) smears, breast cancer screening, and postpartum care. Regularly offered services at the village level were an expectation because the distance from the village to the health care setting posed a major barrier to accessing care, particularly in the rainy season. Emergency mobile clinics were another need of Akha pregnant hill tribe women because when close to delivery, they needed to be able to secure timely access to a hospital, and using their everyday motorcycle was not comfortable and safe for them during late pregnancy. Moreover, the Akha hill tribe women expressed the necessity of basic standard medical equipment at small health-promoting hospitals located at the village level. The Akha women often needed to obtain services at a district hospital for many medical procedures during pregnancy.

A 39-year-old woman said the following [P#53]:

As I am a female, I prefer female staff at the MCH clinic. At least a female understands (another) female, and I can ask her whatever I want to know.

A 28-year-old woman said the following [P#10]:

I want at-home postpartum visit service from health staff. Therefore, I would not need to travel to a city. I cannot drive, and my husband has to work at the farm.

## Discussion

The Akha women who were pregnant or had delivered their infant one year prior and had attended MCH services generally had a poor socioeconomic status. They faced many forms of stigma when attending MCH services in the previous year, such as being spoken to impolitely or with hostility, being physically abused, being refused treatment, and having their personal information intentionally disclosed to the public. Several factors were extracted as the drivers and facilitators of such stigma. Being a member of the Akha people was a stigma marker. Poor rates of accessing MCH services and breast and cervical cancer screening were found among the Akha hill tribe women who needed to access these services. Some people accepted the suffering caused by the stigmatizing experience, while others preferred to ask for better care and service, including seeking care at a private health care provider. Most Akha women expected to have gender-matched health care providers, active and mobile emergency clinics, and full, standard medical equipment at hospitals located in their village. They also wanted an active health care service.

In our study, speaking Akha, wearing traditional dress, being poor, and having tribal names were found to be the drivers of stigma for Akha women when accessing MCH services, especially in public hospitals in Thailand. These factors were very specific stigma scenarios among the Akha hill tribe women when accessing public hospitals in Thailand. These drivers are very difficult to address fully because it is often impossible for Akha people aged 25 years and older to be fluent Thai speakers because they have never been to school and are unable to speak Thai [23]. Changing their traditional dress is also unlikely to be undertaken, since Akha hill tribe women strongly prefer to dress in their own style, which relates to their culture and religion [36]. Moreover, it is very difficult to improve Akha hill tribe family income in the near future given tribe members’ current education status and their nearly universal lack of training in professional skills [30].

All health care providers being Thai, knowledge gaps between health care providers and clients, and gender mismatch with the client were found to be facilitators of stigma. These findings are supported by a study by Nyblade et al. [32], which reported that stigma greatly impacts access to health care services, especially stigma related to different characteristics or backgrounds and knowledge gaps between health caregivers and clients, which eventually lead to poor health outcomes for the population. Many personal characteristics, including the attitudes and behaviors of health care providers, have been reported as facilitators of stigma, especially when directed at vulnerable populations [1, 36, 37].

When attending MCH services in the public hospitals in Thailand, the majority of Akha hill tribes reported experiencing stigma through verbal and physical abuse, a refusal to provide proper treatment and the release of individual information to the public. A high frequency of verbal abuse from health care providers was reported. This point was the strongest and resulted in immense harm to the Akha hill tribe women who attended MCH services in public hospitals. Many questions arose that expressed how this abuse could happen to these people, especially questions regarding why people become nurses or doctors if they will not provide appropriate service or care to people suffering from health problems. The Akha hill tribe women commented that if individuals did not have a health problem, they would not go to a hospital. Verbal abuse from health care providers toward the Akha women made them feel confused about these professionals. Physical abuse, a refusal of treatment and the public sharing of client information were reported in some cases, but none of these should occur in health care settings. Such behavior is contrary to the ethics of health professionals according to the WHO guidelines [38] and the principles of health care ethics [39]. People should receive standard care regardless of their race, tribe, or sociodemographic status.

Due to the stigma encountered by the Akha women when attending an MCH clinic in a public hospital, poor access to the services and poor rates of cervical and breast cancer screening were reported. These findings were supported by a study in two district public hospitals located in the hill tribe villages in northern Thailand, which reported a very poor rate of antenatal care (ANC), cervical and breast cancer screening, and other activities related to women’s reproductive health [40]. In 2018, the Thai Ministry of Public Health [41] reported 8,622 new annual cases of cervical cancer and 5,015 cervical cancer-related deaths among women. In our study, the cervical cancer screening rate among the Akha women was 31.1% compared to 45.6% for Thai people [42].

Most activities at MCH services do not require serious medical attention. Being screened for breast and cervical cancer, given the low rate of these diseases, was not viewed as a serious problem by either clients or health care providers. The lack of regular visits to MCH services during pregnancy also did not have serious consequences, which made the Akha women less concerned and consequently, less likely to engage in activities related to reproductive health and MCH. In addition, given the stigma experienced when accessing MCH services, the patients did not expect to visit a large hospital. The Akha women favored visiting a small hospital in their village. At such hospitals, having standard medical equipment and care at the village level were their main expectations. Moreover, access to MCH services at small hospitals located in their village made accessing them less time-consuming and eliminated the need to travel and other barriers posed by the more complicated process of accessing services in a large, urban hospital. Another expectation was being attended by a gender-matched health care provider when receiving MCH services, which could reduce stigma and improve women’s personal perception of services.

Several limitations were detected in the study. First, although no participants refused to participate in the study or provide information, given the familiarity between the Akha women and the health care workers at the small hospital in their village, many women preferred not to voice too many of their negative experiences with these health care providers. Thus, most of the negative experiences discussed occurred with health care providers who were working in large, urban hospitals. Second, the participants in the study were recruited from seven hill tribe villages, thus, the study primarily presents information and experiences from the Akha people in northern Thailand. In addition, some key informants experienced stigma some time ago, which could have impacted their recall of certain points during the interview and might affect the interpretation. Finally, because the participant selection was primarily executed by the village headmen, it is possible that some people who had experienced serious and direct stigma that is relevant to the research question might not have been selected. Having a clear and careful selection of participants for the study is one of the critical processes in the qualitative method.

## Conclusion

Akha women who attend a public MCH clinic in Thailand suffer stigma driven by their specific characteristics, such as being unable to speak Thai (or lacking fluency), being poor, wearing their traditional dress and using traditional naming conventions. Health care providers’ background, gender mismatches and knowledge gaps between health care providers and clients were identified as facilitators of stigma for women seeking MCH services. Akha women face many forms of stigma when receiving clinical services, including verbal and physical abuse, a refusal to provide treatment, and the intentional disclosure of their personal information to the public. As a result, such mistreatment affects multiple health and health service outcomes, such as poor rates of attending antenatal care and low rates of cervical and breast cancer screening. Akha hill tribe women use many approaches to adjust to the stigma that they encounter, such as accepting the situation with no better option, defending themselves to obtain better care and services, and using a private clinic instead. Akha women expect to have active services and gender-matched health care providers at MCH services and mobile emergency clinics and that the appropriate equipment be provided to equip standard MCH services at hospitals located in or near the hill tribe villages.

Serious consideration of the problems posed by the stigmatization for Akha hill tribe women is needed to improve their access to health care services, particularly those attending MCH services. Policies with standard protocols to ensure the provision of equal care for everyone should be implemented. The improvement of health facilities located in hill tribe villages should also be considered to familiarize hill tribe people with health care providers in their home setting. Moreover, encouraging hill tribe people to be trained as nurses or medical doctors and then sending them back to work at a hospital in their village could eventually reduce stigma.

## Electronic supplementary material

Below is the link to the electronic supplementary material.

Supplementary Material 1

Supplementary Material 2

## Data Availability

The datasets used and/or analyzed during the current study are available from the corresponding author on reasonable request.

## List of abbreviations

ANC: Antenatal care
HIV: Human immunodeficiency virus
ID: Identification card.
MCH: Maternal and child health care.
PAP: Papanicolaou test.
WHO: World Health Organization

## References

[1] Knaak S, Szeto A (2017). Mental illness-related stigma in healthcare barriers to access and care and evidence-based solutions. Healthc Manage Forum.

[2] Goldenberg T, Jadwin-Cakmak L, Popoff E, Reisner SL, Campbell BA, Harper GW (2019). Stigma, gender affirmation, and primary healthcare use among black transgender youth. J Adolesc Health.

[3] Kim HY, Grosso A, Ky-Zerbo O, Lougue M, Stahlman S, Samadoulougou C (2018). Stigma as a barrier to health care utilization among female sex workers and men who have sex with men in Burkina Faso. Ann Epidemiol.

[4] Arnaez JM, Krendl AC, McCormick BP, Chen Z, Chomistek AK (2020). The association of depression stigma with barriers to seeking mental health care: a cross-sectional analysis. J Mental Health.

[5] Stangl AL, Earnshaw VA, Logie CH, Brakel W, Simbayi LC, Barré I (2019). The health stigma and discrimination framework: a global, crosscutting framework to inform research, intervention development, and policy on health-related stigmas. BMC Med.

[6] Crumb L, Mingo TM, Crowe A (2019). “Get over it and move on”: the impact of mental illness stigma in rural, low-income United States populations. Mental Health & Prevention.

[7] World Health Organization (WHO). Maternal health. Available from: https://www.who.int/health-topics/maternal-health#tab=tab_1. Assessed 04 august 2021.

[8] World Health Organization (WHO). Maternal health. Available from: https://www.who.int/en/news-room/fact-sheets/detail/maternal-mortality. Assessed 04 august 2021.

[9] Nayar US (2014). Reducing stigma and discrimination to improve child health and survival in low-and-middle income countries: promising approaches and implications for future. J Health Communication.

[10] Ranji U, Long M, Salganicoff A. Beyond the numbers: access to reproductive health care for low-income women in five communities. Available from: https://files.kff.org/attachment/Executive-Summary-Beyond-the-Numbers-Access-to-Reproductive-Health-Care-for-Low-Income-Women-in-Five-Communities. Assessed 04 august 2021.

[11] Wado YD (2018). Women’s autonomy and reproductive health-care-seeking behavior in Ethiopia. Women & Health.

[12] United States Agency for International Development (USAID). Stigma and discrimination: key barriers to achieving global goals for maternal health and elimination of new child HIV infection. Available form: https://www.healthpolicyproject.com/pubs/92_WorkingPaperStigmaPMTCTJuly.pdf Accessed 27 February 2022.

[13] World Health Organization (WHO). Revised programme budget 2020–2021 human reproduction programme (HRP). Available from: https://apps.who.int/iris/rest/bitstreams/1304356/retrieve.Assessed 05 august 2021.

[14] Schäferhoff M, Hoog SV, Martinez S, Fewer S, Yamey G. Funding for sexual and reproductive health and rights in low- and middle-income countries: threats, outlook, and opportunities. Available from: https://www.who.int/pmnch/media/news/2019/srhr_forecast_web.pdf.Assessed 05 august 2021.

[15] Panichkriangkrai W, Topothai C, Saengruang N, Thammatach-Aree J, Tangcharoensathien V (2020). Universal access to sexual and reproductive health services in Thailand: achievements and challenges. Sex Reproductive Health Matters.

[16] Hennegan J, Winkler IT, Bobel C, Keiser D, Hampton J, Larsson G (2021). Menstrual health: a definition for policy, practice, and research. Sex Reproductive Health Matters.

[17] Minnis AM, Krogstad E, Quinn MKS, Agot K, Ahmed K, Wagner LD (2021). Giving voice to the end-user: input on multipurpose prevention technologies from the perspectives of young women in Kenya and South Africa. Sex Reproductive Health Matters.

[18] Oldertrøen KS, Boer M, Nyheim KS, Thoresen L. Male partners’ experiences of caregiving for women with cervical cancer - a qualitative study. Journal of Clinical Nursing. 2019; 28(5–6): 987 – 96. DOI: 10.1111/jocn.14688. 10.1111/jocn.14688 30302850

[19] Apidechkul T, Laingoen O, Suwannaporn S (2016). Inequity in accessing health care service in Thailand in 2015: a case study of the hill tribe people in Mae Fah Luang district, Chiang Rai, Thailand. J Health Res.

[20] Kumpalanon J, Ayuwat D, Sanchaisuriya P (2012). Developing of health promoting of district hospitals in Thailand. Am J Health Sci.

[21] Ministry of Public Health, Thailand. Guideline for MCH care. Available from: https://hp.anamai.moph.go.th/th/mch-emag/203656.

[22] Ruangrit P, Jantakat N, Chemplee K, Pattanaporn K, Sopajaree C, Apidechkul T (2021). Maternal and child health system for the hill tribe in northern Thailand: outcomes and barriers. J Health Sci Altern Care.

[23] Apidechkul T, Wongnuch P, Sittisarn S, Ruanjai T (2016). Health situation of Akha hill tribe in Chiang Rai province, Thailand. J Public Health Dev.

[24] Marques P, Gama A, Santos M, Heleno B, Vermandere H, Dias S (2021). Understanding cervical cancer screening barriers among migrant women: a qualitative study with healthcare and community workers in Portugal. Int J Environ Res Public Health.

[25] Rocha TJ, Morales SM, Fernández CC, Brouwer KC, Goldenberg SM (2018). Stigma and unmet sexual and reproductive health needs among international migrant sex workers at the Mexico–Guatemala border. Int J Gynecol Obstet.

[26] Hussein J, Ferguson L (2019). Eliminating stigma and discrimination in sexual and reproductive health care: a public health imperative. Sex Reproductive Health Matters.

[27] Princess Maha Chakri Siridhorn Anthropology center. Hill tribe. 2020. Available from: http://www.sac.or.th/main/index.php. Assessed 10 august 2021.

[28] Singkhorn O, Apidechkul T, Pitchalard K, Moonpanane K, Hamtanon P, Sunsern R (2021). Prevalence of and factors associated with depression in the hill tribe population aged 40 years and older in northern Thailand. Int J Mental Health Syst.

[29] Apidechkul T (2018). Prevalence and factors associated with type-2 diabetes mellitus and hypertension among the hill tribe elderly populations in northern Thailand. BMC Public Health.

[30] Apidechkul T, Tamornpark R, Chomchoei C, Upala P, Yeemard F (2021). Association between lifestyle behaviors and hypertension among hill tribe adults in Thailand: a cross-sectional study. J Racial Ethnic Health Disparities.

[31] Ong PA, Seangpraw K (2019). Association between self-care behaviors and quality of life among elderly minority groups on the border of Thailand. J Multidisciplinary Healthc.

[32] Nyblade L, Stockton MA, Giger K, Bond V, Ekstrand ML, Mc Lean R (2019). Stigma in health facilities: why it matters and how we can change it. BMC Med.

[33] Jacoby A (1994). Felt versus enacted stigma: a concept revisited: evidence from a study of people epilepsy in remission. Social Science& Medicine.

[34] Alhazmi AA, Kaufmann A (2022). Phenomenological qualitative methods applied to the analysis of cross-cultural experience in novel educational social contexts. Front Psychol.

[35] World Medical Association. Declaration of Helsinki: Ethical principles for medical research involving human subjects. Available from: https://www.med.or.jp/dl-med/wma/helsinki2013e.pdf. Assessed 04 august 2021. 15835069

[36] Owuor JO. Internalized stigma as a barrier to access to health and social care services by minority ethnic groups in the UK. Available from: https://raceequalityfoundation.org.uk/wp-content/uploads/2018/02/Health-Briefing-36_1.pdf. Accessed 22 August 2021.

[37] Department of Disease Control. Ministry of Public Health, Thailand. Stigma and discrimination among health care providers and people living with HIV in health care settings in Thailand: comparison of finding from 2014–2015 and 2017. Available from: https://hivhub.ddc.moph.go.th/Download/Report/S_D/2_UNAIDS_Final_S_D_Final_Health_care_settings_Comparison_a5.pdf. Accessed 22 August 2021.

[38] World Health Organization (WHO). Code of ethics and professional conduct. Available from: https://www.who.int/about/ethics/code_of_ethics_abridged.pdf. Accessed 23 August 2021.

[39] Summers J. Principles of healthcare ethics. Available from: http://samples.jbpub.com/9781449665357/chapter2.pdf. Accessed 23 August 2021.

[40] Ruangrit P, Jantakat N, Chimplee K, Pattanaporn K, Sopajaree C, Apidechkul T (2021). Maternal and child health services for the hill tribe and stateless population in northern Thailand: outcomes and barriers. J Health Sci Altern Med.

[41] Ministry of Public Health. Thailand. Thailand human papillomavirus and related cancers. Available from: https://hpvcentre.net/statistics/reports/THA_FS.pdf. Accessed 23 August 2021.

[42] Sorotkulangkoon P, Thato R (2019). Factors predicting cervical cancer screening among police officer wives in Bangkok. J Police Nurse.

