# Supplementary material for: Components of stigma and its impact on maternal and child health service and outcomes: perspective of Akha hill tribe women in Thailand
Source: BMC Health Serv Res. 2022 Oct 19;22:1263. doi: 10.1186/s12913-022-08622-x (PMC9583464; doi:10.1186/s12913-022-08622-x)
Supplement: Supplementary file 1 — Supplementary Material 1 [file 12913_2022_8622_MOESM1_ESM.docx]

**Question guide**

1) Which hospital did you attend for MCH?

2) Did you experience any discomfort or stigma when attending MCH?

3) Could you provide information in terms of frequency, who displayed stigmatizing behaviors, and in what form?

4) How did you feel about this experience?

5) How did you respond to these behaviors?

6) What is your expectation about accessing MCH?

7) Did you experience other barriers to accessing MCH?
