# Supplementary material for: Components of stigma and its impact on maternal and child health service and outcomes: perspective of Akha hill tribe women in Thailand
Source: BMC Health Serv Res. 2022 Oct 19;22:1263. doi: 10.1186/s12913-022-08622-x (PMC9583464; doi:10.1186/s12913-022-08622-x)
Supplement: Supplementary file 2 — Supplementary Material 2 [file 12913_2022_8622_MOESM2_ESM.docx]

**Consolidated criteria for reporting qualitative studies (COREQ): 32-item checklist**

| **Topic and item No.** | **Guide Questions/Description** | **Reported on Page No.** |
| --- | --- | --- |
| **Domain 1: research team and reflexivity** | |  |
| Personal Characteristics |  |  |
| 1. Interviewer/facilitator | Which author/s conducted the interview or focus group? | Page 5 |
| 2. Credentials | What were the researcher’s credentials? E.g. PhD, MD | Page 5 |
| 3. Occupation | What was their occupation at the time of the study? | Page 5 |
| 4. Gender | Was the researcher male or female? | Page 5 |
| 5. Experience and training | What experience or training did the researcher have? | Page 5 |
| Relationship with participants | |  |
| 6. Relationship established | Was a relationship established prior to study commencement? | Page 5 |
| 7. Participant knowledge of the interviewer | What did the participants know about the researcher? *e.g. personal goals, reasons for doing the research* | Page 5 |
| 8. Interviewer characteristics | What characteristics were reported about the interviewer/facilitator? e.g. *Bias, assumptions, reasons and interests in the research topic* | Page 5 |
| **Domain 2: study design** | |  |
| Theoretical framework |  |  |
| 9. Methodological orientation and Theory | What methodological orientation was stated to underpin the study? *e.g. grounded theory, discourse analysis, ethnography, phenomenology, content analysis* | Page 4 |
| Participant selection | |  |
| 10. Sampling | How were participants selected? *e.g. purposive, convenience, consecutive, snowball* | Page 4-5 |
| 11. Method of approach | How were participants approached? *e.g. face-to- face, telephone, mail, email* | Page 5 |
| 12. Sample size | How many participants were in the study? | Page 5 |
| 13. Non-participation | How many people refused to participate or dropped out? Reasons? | No |
| Setting | |  |
| 14. Setting of data collection | Where was the data collected? *e.g. home, clinic, workplace* | Page 4 |
| 15. Presence of non-participants | Was anyone else present besides the participants and researchers? | no |
| 16. Description of sample | What are the important characteristics of the sample? *e.g. demographic data, date* | Page 5 |
| Data collection | |  |
| 7. Interview guide | Were questions, prompts, guides provided by the authors? Was it pilot tested? | Page 4 |
| 18. Repeat interviews | Were repeat interviews carried out? If yes, how many? | No |
| 19. Audio/visual recording | Did the research use audio or visual recording to collect the data? | Page 5 |
| 20. Field notes | Were field notes made during and/or after the interview or focus group? | Page 5 |
| 21. Duration | What was the duration of the interviews or focus group? | Page 5 |
| 22. Data saturation | Was data saturation discussed? | Page 5 |
| 23. Transcripts returned | Were transcripts returned to participants for comment and/or correction? | Page 5 |
| **Domain 3: analysis and findings** | |  |
| Data analysis | |  |
| 24. Number of data coders | How many data coders coded the data? | Page 5 |
| 25. Description of the coding tree | Did authors provide a description of the coding tree? | Page 5 |
| 26. Derivation of themes | Were themes identified in advance or derived from the data? | Page 5 |
| 27. Software | What software, if applicable, was used to manage the data? | Page 5 |
| 28. Participant checking | Did participants provide feedback on the findings? | Page 5 |
| Reporting | |  |
| 29. Quotations presented | Were participant quotations presented to illustrate the themes / findings? Was each quotation identified? *e.g. participant number* | Page 7-15 |
| 30. Data and findings consistent | Was there consistency between the data presented and the findings? | 5-15 |
| 31. Clarity of major themes | Were major themes clearly presented in the findings? | 5-15 |
| 32. Clarity of minor themes | Is there a description of diverse cases or discussion of minor themes? | 5-15 |
